# 

**Published:** 1880-02

**Authors:** 


### CONTENTS:

ARTICLE I.—Proceedings of the Maryland and District of Columbia Dental Society. (Continued.)........ 433

ARTICLE II.—Diseases of the Mouth. Review of Dr. Sexton's Article. By Prof. Hodgkin........ 447

ARTICLE III.—Alveolar Abscess. By John H. Coyle, D. D. S........ 455

ARTICLE IV.—Cleaning Teeth. By Dr. G. A. Mills........ 460

ARTICLE V.—Dr. Waters' Statement of the Gardner Case........ 464

ARTICLE VI.—Abrasion of the Teeth. By Dr. Hermann........ 468

ARTICLE VII.—Tooth-ache as a Cause of Paralysis. By T. H. Parramore, D. D. S.. 470

ARTICLE VIII.—American Academy of Dental Science........ 472

### EDITORIAL, ETC.

Bills to Regulate the Practice of Dentistry in Maryland and District of Columbia.... 473

Dentistry in the District of Columbia........ 476

Honors to American Physicians........ 477

### MONTHLY SUMMARY.

Treatment of Fistulæ, and Scars of the Cheek........ 478

Effects of Arsenic on the Body........ 479

Death from a Tooth in the Trachea........ 480

Saliva and the Digestion of Starch........ 480

Success of Different Treatment of Typhoid Fever........ 480

A Question........ 480

Arsenic in Bismuth........ 480

